# The Educational Alliance: Essential to Implement Competency-Based Health Professions Education

**DOI:** 10.5334/pme.2607

**Published:** 2026-09-09

**Authors:** Karen E. Hauer, Mary Ellen J. Goldhamer, Jonathan Amiel, Denyse Richardson, Alan Schwartz, Adrian P. Marty, Laura Molgaard, Severin Pinilla, Jan Breckwoldt, As members of the International Competency-Based Health Professions Educators Collaborative

**Affiliations:** 1 Department of Medicine, University of California, San Francisco (UCSF) School of Medicine, San Francisco, CA, USA; 2 Harvard Kidney Urology Hematology Training Institute, Boston, Massachusetts, USA; 3 New York Presbyterian, New York, NY, USA; 4 Stanford University, Palo Alto, CA, USA; 5 Department of Medicine, University of Alberta, Edmonton, Alberta, Canada; 6 Department of Medical Education, University of Illinois Chicago, Chicago, Illinois, USA; 7 Department of Pediatrics, University of Illinois Chicago, Chicago, Illinois, USA; 8 University of Illinois Chicago School of Law, Chicago, Illinois, USA; 9 Department of Anesthesiology, Intensive Care Medicine and Pain Therapy, University Hospital Balgrist, Zurich, Switzerland; 10 University of Minnesota College of Veterinary Medicine, St. Paul, Minnesota, USA; 11 University Hospital of Old Age Psychiatry and Psychotherapy, University of Bern, Bern, Switzerland; 12 Institute of Anaesthesiology and Perioperative Medicine, University Hospital Zurich, University of Zurich, Zurich, Switzerland

## Abstract

The educational alliance describes a framework for the trusting relationship between trainee and supervisor as they engage in meaningful, effective feedback. This framework emphasizes interpersonal relationships, shared goals, and agreement on actionable steps to achieve these goals. In this theory-informed conceptual analysis, the authors propose that the educational alliance logically extends to support and enhance competency-based medical education and health professions education, collectively known as competency-based health professions education (CBHPE). Authors describe how key features of the educational alliance align with the five core components of CBHPE: outcome-based competencies, sequenced progression, tailored learning experiences, competency-focused instruction, and programmatic assessment – by facilitating individualized learning that is centered on the trainee-supervisor relationship. Relevant theories that support the effective implementation of the educational alliance in CBHPE to advance learning include growth mindset and self-regulated learning theories. Authors present specific actionable recommendations regarding trainee and supervisor roles and responsibilities within an educational alliance for creating shared mental models, building relationships, engaging in team training, and attending to ethical considerations, including conflicts of interest and using data for the determination of trainee competency and advancement. Additionally, the authors introduce an Educational Agreement applicable to all training levels, designed to foster, guide, and strengthen the educational alliance. The Agreement is intended to be voluntary, co-constructed, and may be adapted to local contexts. Finally, the authors identify areas for future empirical examination through research on the educational alliance in CBHPE programs.

## Introduction

Competency-based health professions education (CBHPE) prioritizes trainees actively participating in their own development through self-regulated learning, engagement in assessment as a source of feedback for learning, and personal reflection on their experiences and learning progress. Essential to the success of CBHPE is a partnership between trainee and supervisor to share responsibility for the trainee’s growth, with the trainee’s trust in the intent and usefulness of the faculty’s feedback [1]. The educational alliance (Figure 1) has been identified as a potentially mediating element in CBHPE, fundamentally shaping the quality and effectiveness of feedback for learning [2]. Conceptualizations of feedback have evolved from the one-way delivery of information from trainee (used here synonymously with student, resident, fellow, learner) to supervisor (teacher, clinical teacher, faculty) toward a dialogue in which feedback effectiveness depends on relationship, context, content, delivery, and receipt of feedback [3]. Ideally, both the trainee and the supervisor demonstrate feedback literacy (seeking, interpreting, acting upon, and sharing feedback) to promote the trainee’s competency-based development through iterative assessment, coaching, and progression decisions central to CBHPE [4,5].

**Figure 1:**
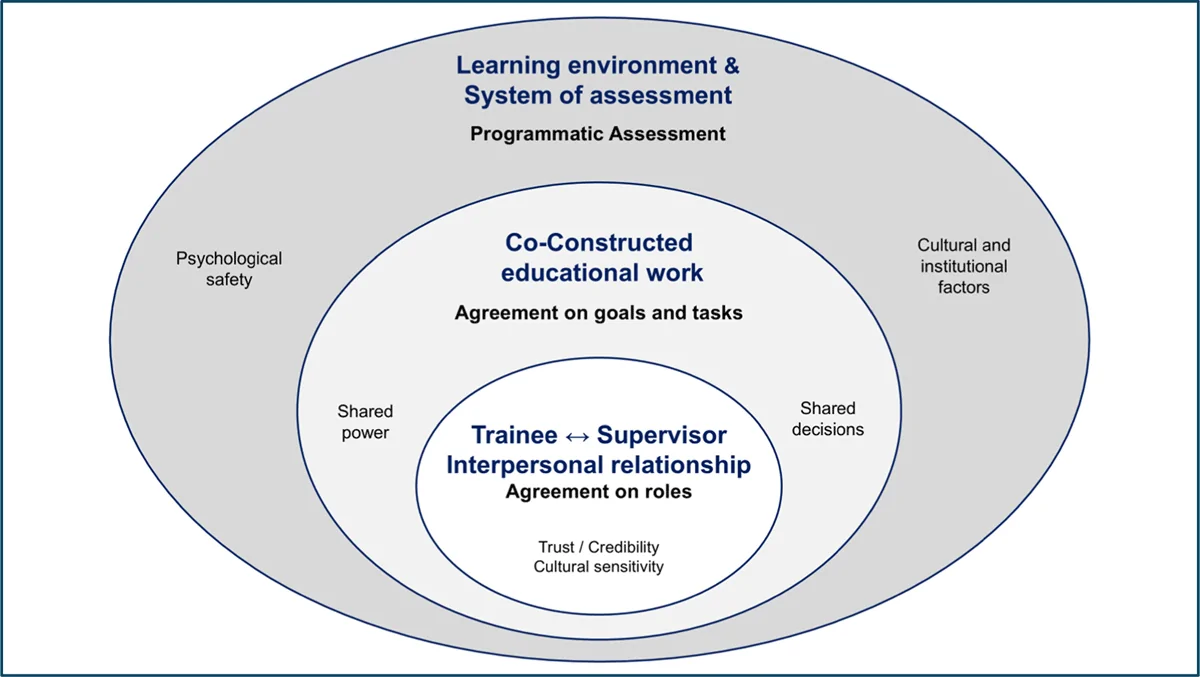
**Dimensions of the Educational Alliance in Competency-Based Education**. The figure depicts three interrelated layers: (center) the relational foundation of mutual trust and respect; (middle) shared goals and tasks that structure the collaborative educational process; and (outside) the contextual environment - including programmatic assessment systems, psychological safety and institutional culture - that shapes and is shaped by the alliance. The three layers of the figure symbolize reciprocal influence and feedback loops representing dialogue, reflection, and adaptive calibration across the layers.

Telio defined the educational alliance as a trusting relationship between trainee and supervisor that constitutes the context for effective feedback [2]. The educational alliance comprises a relational bond, shared goals, and agreement on tasks to achieve goals [2]. Whereas the educational alliance has been proposed to facilitate rich feedback discussions between trainee and supervisor, little evidence or discussion exists to date to support theory-informed benefits for CBHPE by supporting not only tailored feedback but also personalized and relationship-centered learning and a growth mindset. The educational alliance can facilitate trainees’ and supervisors’ trust in each other and their mutual trust in competency-based assessment systems and outcomes [6].

The purpose of this manuscript, a theory-informed conceptual analysis, is to elaborate on the potential benefits of the educational alliance as a mechanism for implementing the core components of CBHPE, outlined below [7]. Authors first review the conceptual foundations of the educational alliance, its grounding in the therapeutic alliance, and its theoretical relevance to CBHPE. Description of the potential of the educational alliance to drive achievement of the core components of CBHPE and boundaries of the alliance set the stage for recommendations to maximize the impact of the educational alliance in CBHPE programs. Taken together, this expanded understanding can inform efforts to facilitate educational alliances within CBHPE programs that prepare trainees to become health practitioners who engage deeply in learning activities to achieve program outcomes and continue to learn through their careers.

## Methods

Manuscript authors, including experienced health professions educators and researchers, are all members of the International Competency-Based Health Professions Educators Collaborators (ICBHPE). They developed this manuscript beginning with a narrative literature review on the topics of the educational alliance, therapeutic alliance, feedback dialogue and feedback literacy, and programmatic assessment in CBHPE. At a day-long, international, in-person summit, authors compared and contrasted the educational and therapeutic alliance, discussed the enthusiasm in the literature for the educational alliance to strengthen feedback and elaborated on its unexplored potential to strengthen multiple aspects of CBHPE. Through monthly virtual meetings, they explored the theoretical underpinnings of the educational alliance and generated recommendations to strengthen it within CBHPE programs, drawing on the literature and their experience as educators and education program leaders. They resolved disagreements through discussion and challenged one another to identify boundaries of what is known or unanswered about the educational alliance.

### Contrasting Educational Alliance and Therapeutic Alliance

The educational alliance was adapted from the concept of the therapeutic alliance, also known as the working alliance, between client (patient) and psychotherapist [8,9,10]. The therapeutic alliance is one of the most important determinants of therapeutic success [11,12], with empirical research demonstrating beneficial patient/client outcomes and strengthening evidence about its nature, including how it can be measured and understood [13,14,15,16].

Extending this relational framing to the educational alliance dyad in CBHPE (Table 1), trust and shared power remain central. This alliance represents a relationship built upon three essential elements: the development of interpersonal connections (interpersonal bond enabling psychological safety necessary for honest assessment and uptake of feedback) [2,17], mutual understanding of the goals of the relationship and its boundaries (terms of engagement - clarifying expectations around competencies, assessment standards, and progression), and agreement on the goals and methods of the relationship (task orientation aligning supervisor–trainee activities with competency development, workplace-based assessment, and evidence used for progression decisions). In CBHPE, where assessment is continuous and progression decisions are consequential, the quality of the educational alliance may shape both the credibility and use of assessment information.

**Table 1:** Key features of the educational alliance, alignment with the five core components of competency-based education, and the responsibilities of trainees and supervisors to promote a successful alliance for learning.


FEATURE OF THE EDUCATIONAL ALLIANCE	EXPLANATION IN COMPETENCY-BASED EDUCATION PROGRAM, TIED TO CORE COMPONENTS [7]	TRAINEE RESPONSIBILITY/ACTION	SUPERVISOR RESPONSIBILITY/ACTION

**Interpersonal Relationship**			

Mutual respect	Tailored learning experiences that facilitate the developmental acquisition of competency	Contribute to a culture that encourages supervisors to provide honest and actionable feedback, and trainees to engage in feedback dialogues Trust in formative feedback that is intended to enhance learning and skill building	Create a culture of trust and psychological safety Foster cultural humility Attention to professional identity formation

**Goals of engagement**			

Shared purpose and goals	Outcome competencies	Co-production of learning goals aligned with the outcome competencies	

Shared agreement on how trainees and supervisors will interact	Tailored learning experiences that facilitate the developmental acquisition of competency	Shared agreement between supervisor and trainee to dedicate time for goal setting, direct observation, feedback, coaching	

Mutual accountability for learning outcomes	Outcome Competencies	Collaborative goal setting to include iterative updates to individualized learning plans and a realistic timeline for achievement of competencies	

Commitment to honest feedback and continuous improvement	Competency focused instruction to promote developmental acquisition of competency	Engage in self-reflective practice, seek and accept feedback, embrace a growth mindset rather than a performance mindset	Coaching methods to promote feedback, dialogue, and growth (R2C2 coaching [48]) Engage in relevant faculty development including coaching theory and methods Encourage trainee to adopt a growth mindset, avoidance of a false growth mindset

**Task orientation (how to pursue agreed-upon goals)**			

Shared identification of learning needs and opportunities	Outcome Competencies	Co-produce learning goals and individualized learning plans that include an action plan	

Shared focus on feedback	Competency focused instruction to promote developmental acquisition of competency	Recognize the value in self-reflective practice, seek and accept feedback, embrace a growth mindset rather than performance mindset	Coaching methods to promote feedback, dialogue, and growth (R2C2 coaching) Seek peer feedback and faculty development opportunities to grow as a supervisor

Alignment on assessment methods and criteria	Programmatic Assessment	Seek to understand the formative and summative criteria and assessments that inform competency-based advancement	Ensure trainee understands the formative and summative criteria and assessments that inform competency-based advancement

Engagement in reflection	Tailored learning experiences to facilitate the developmental acquisition of competency	Recognize the value of self-assessment informed by feedback and multi-source work-place based assessments	Reflect on the supervisor role in facilitating trainee self-assessment to promote growth Encourage trainee to incorporate feedback and multi-source evaluations in their self-assessment along with reflective practice

Cultivation of trainee’s agency for self-direction	Competency-focused instruction informs teaching practices that promote the developmental acquisition of competency	Compare self-assessment with supervisor feedback Iteratively update learning goals and individualized learning plan	Encourage trainee self-assessment with structured feedback conversations to inform progress


Beyond their similarities, the therapeutic alliance in psychotherapy also has notable distinctions from the educational alliance. The therapeutic alliance is typically a protected, exclusive, and dyadic relationship that fosters trust and introspection, free from external professional influences. In contrast, the educational alliance in HPE, while also grounded in trust and guidance, unfolds within a complex educational and team-based workplace ecosystem where some observations and information are available to and shared with others. Supervisory relationships in clinical workplaces are shaped by the dynamics of the interprofessional team, the trainee’s peer group, experiences with patients, and clinical environment affordances and constraints. Both trainee and supervisor can strive to counteract these contextual factors and build a robust educational alliance by communicating openly, setting clear expectations, and creating spaces for reflection.

### Theories Relevant to Educational Alliance and Competency-Based Health Professions Education

Though rooted in therapeutic alliance concepts from the 1950s, the educational alliance also aligns with more contemporary educational theories, which unmask how the alliance could serve as a crucial facilitating mechanism for CBHPE. This section elaborates on select theories of particular relevance: growth mindset theory [18] and self-regulated learning theory [19]. Understanding these theoretical foundations supports successful CBHPE implementation by explicating the mechanisms through which the educational alliance can provide the relational foundation necessary for trainees and supervisors to navigate competency-based development and challenges effectively. Authors acknowledge that other theoretical lenses, including sociocultural and situated learning theories, are also relevant to situate the educational alliance within broader clinical learning environments. Growth mindset and self-regulated learning are foregrounded because they most directly illuminate mechanisms through which the alliance operates at the trainee–supervisor level as learners engage with feedback, regulate their learning, and persist through iterative assessment in CBHPE.

Growth mindset theory suggests that learning capacity can be developed through effort, effective strategies, and guidance from others [18]. Accordingly, abilities and intelligence are not fixed traits but can be developed through dedicated learning. These tenets align with the educational alliance’s emphasis on collaborative goal setting and feedback [2]. Within CBHPE, a growth mindset may function as a mechanism that increases trainees’ willingness to seek, accept, and act on formative assessment data, thereby enhancing the use of feedback to inform competency development and progression. Individuals manifesting a growth mindset believe they can improve through engaged, targeted practice. A trusted supervisor, such as within an educational alliance, reinforces these beliefs and fosters a growth mindset through their approach to feedback. The educational alliance and CBHPE both promote achieving competencies through iterative practice, observation, and feedback (e.g., through work-based assessments) rather than merely completing courses or time-based training programs [20]. Supervisors can foster a growth mindset in trainees through praising effort and learning strategies and processes, encouraging the embracement of challenges, normalizing setbacks or failures, and expressly modeling a growth mindset. In a CBHPE program, educators encourage reflective learning and practice, providing guidance, feedback, and resources that help trainees progress towards competency goals. Supervisors who themselves adopt a growth mindset and model feedback seeking, reflection, and lifelong learning foster the psychological safety and trustworthiness that underpin the educational alliance [20].

Self-regulated learning theory emphasizes that trainees, regardless of stage of training, actively manage their own learning [19]. This concept is foundational to CBHPE [21]. Self-regulated learning skills involve setting learning goals, monitoring one’s progress, and adjusting or adapting strategies to advance development. In CBHPE, self-regulated learning operates as a mechanism through which trainees interpret and integrate multiple assessment inputs, set competency-aligned goals, and adjust strategies in response to feedback and performance data. The self-regulated learning process is not linear, but rather requires iterative cycles of planning, enacting, reflecting and adjusting thoughts and actions to achieve goals [22]. Developing self-regulated learning skills cultivates adaptive, lifelong learners who are creative yet critical thinkers and problem solvers able to work and learn both independently and collaboratively [23]. To develop and practice self-regulated learning skills, supervisors need to foster an environment that enables taking learning risks and learning from both successes and failures. An established educational alliance empowers trainees to proactively engage in their education and continuous development. An educational alliance simultaneously facilitates self-regulated learning by affording guidance for setting realistic yet appropriately challenging learning goals and developing effective plans throughout the learning process.

Taken together, growth mindset and self-regulated learning represent inherently fragile constructs requiring ongoing reinforcement. The educational alliance provides this scaffolding via its collaborative trainee-supervisor relationship that facilitates trainees’ development toward autonomous professional competence.

### The Importance of the Educational Alliance for Advancing the Core Components of Competency-Based Health Profession Education

Literature to date on the educational alliance in HPE has focused almost exclusively on supervisor-trainee feedback [2,24,25,26]. However, conceptual benefits of the educational alliance extend to all five core components of CBHPE: outcome-based competencies, sequenced progression, tailored learning experiences, competency-focused instruction, and programmatic assessment [7] (Table 1).

The first core component of CBHPE, clearly defined *outcome competencies*, is strengthened by the educational alliance so that expectations may be jointly understood and co-owned within a trusting supervisor-trainee relationship. The second core component, *progressive sequencing of competencies*, means that learning experiences, performance expectations, and assessments are deliberately structured to build sequentially. Trainees advance from novice to competent through an intentional continuum of increasing complexity, responsibility, and removal of supervision. The educational alliance supports this progressive sequencing through a shared mental model of progression, co-constructed by trainee and supervisor. The supervisor helps make the developmental trajectory explicit, and the trainee can drive analysis of how their current performance fits into the larger continuum. The alliance builds trust that feedback supports progressive growth rather than being a punitive judgment [4]. As responsibilities and expectations increase, trainees need to take risks, seek constructive feedback, reflect upon their own performance, and sometimes experience setbacks. A strong alliance empowers trainees to assess their readiness, articulate goals, and advocate for individualized next steps within the competency-based education framework. The longitudinal nature of an educational alliance enables trainees and supervisors to track performance together over time, enabling trainees to seek and integrate experiences and advance their competence coherently [27,28].

The educational alliance facilitates the third CBHPE core component, *tailored learning experiences*, as the dyad co-creates individual opportunities to meet trainees’ current abilities and next steps. Trainees, in turn, can trust their supervisor’s expectations about their next appropriate actions [29]. Similarly, the fourth core component, *competency-focused instruction*, benefits from the educational alliance where supervisors understand the competencies and the trainee’s current strengths and limits. For example, in a longitudinal integrated clerkship model, students perceive their preceptors sharing mutual goals for student learning, and preceptors in turn describe tailoring instruction to students’ developmental state throughout the year [30,31]. As another example, the Promotion in Place training model affords a period of ‘sheltered independence’ with learning experiences tailored to qualifying graduates’ interests and future needs as they transition to fellowship or unsupervised practice [32,33]. In nursing education, a CBHPE nursing program enables self-pacing to meet workforce needs by accelerating some trainees into practice based on readiness [34].

The educational alliance supports the fifth component, *programmatic assessment*, which requires frequent observations for assessment of trainees by supervisors, often including trainee-initiated assessment processes [35]. Feedback is a building block of programmatic assessment, with each feedback event (observation, test, encounter) informing the trainee about their performance and growth. However, trainees are emotionally vulnerable when soliciting and receiving feedback and must trust supervisors’ beneficent intent to gain from the feedback process [29]. A strong educational alliance facilitates this trust, creating the relational context in which feedback is both delivered and actively sought. This context extends beyond the decontextualized ‘rules’ of effective feedback (i.e., feedback should be timely, specific, based on direct observation, actionable) to allow frequent, high-quality feedback to be perceived as credible, co-owned, and supporting goals [2,36]. Thus, the educational alliance serves as a cornerstone of effective competency-based educational practice.

In summary, a strong educational alliance, as conceptualized in the literature to date, ensures that the processes of learning, teaching, and demonstrating competence are jointly understood, contextualized to the trainee’s stage, and actively co-produced. By promoting engagement in learning, the alliance facilitates trainees’ ownership of their development and motivation in the face of challenges [37]. This engagement is not limited to discrete tasks but extends to a general orientation toward lifelong learning and professional identity formation. Within CBHPE, where progression depends on trainees’ active ownership of and participation in assessment and feedback processes, the alliance has been described as a critical factor in sustaining motivation and supporting the developmental trajectory across sequenced competencies [38,39]. When trainees perceive a strong alliance with faculty coaches or supervisors, they are more likely to trust the assessment system, engage with programmatic assessment data, and use feedback constructively [40,41,42]. Without the alliance, CBHPE risks becoming a set of checklists and requirements to be tolerated or feared; with the alliance, CBHPE becomes a personalized, trust-based approach that genuinely supports competence development. Thus, the educational alliance is a working relationship, continuously negotiated, that underpins the relational aspects of CBHPE and also broader trainee engagement required for successful competency development and ongoing learning.

### An Educational Agreement May Guide and Strengthen the Educational Alliance

To foster, guide, and strengthen the educational alliance between trainee and supervisor, authors suggest implementing an Educational Agreement and present a constructed example tool, intended to support dialogue and promote a trusting relationship (Chart). The Agreement can be flexibly adapted to meet the needs of local CBHPE contexts and dyads and to promote and enhance conversations that result in a productive relationship. Dyads are encouraged to develop a shared understanding of how they will proceed together through each category of the agreement. Suggested trainee and supervisor roles for each feature of the educational alliance are outlined in Table 1 and may be used as a template or reference while completing the agreement. “Sentence starters,” shown in the Agreement, facilitate trainee-supervisor conversations with the goal of co-constructing an effective educational alliance to support the trainee’s competency-based progression.

**Chart:**
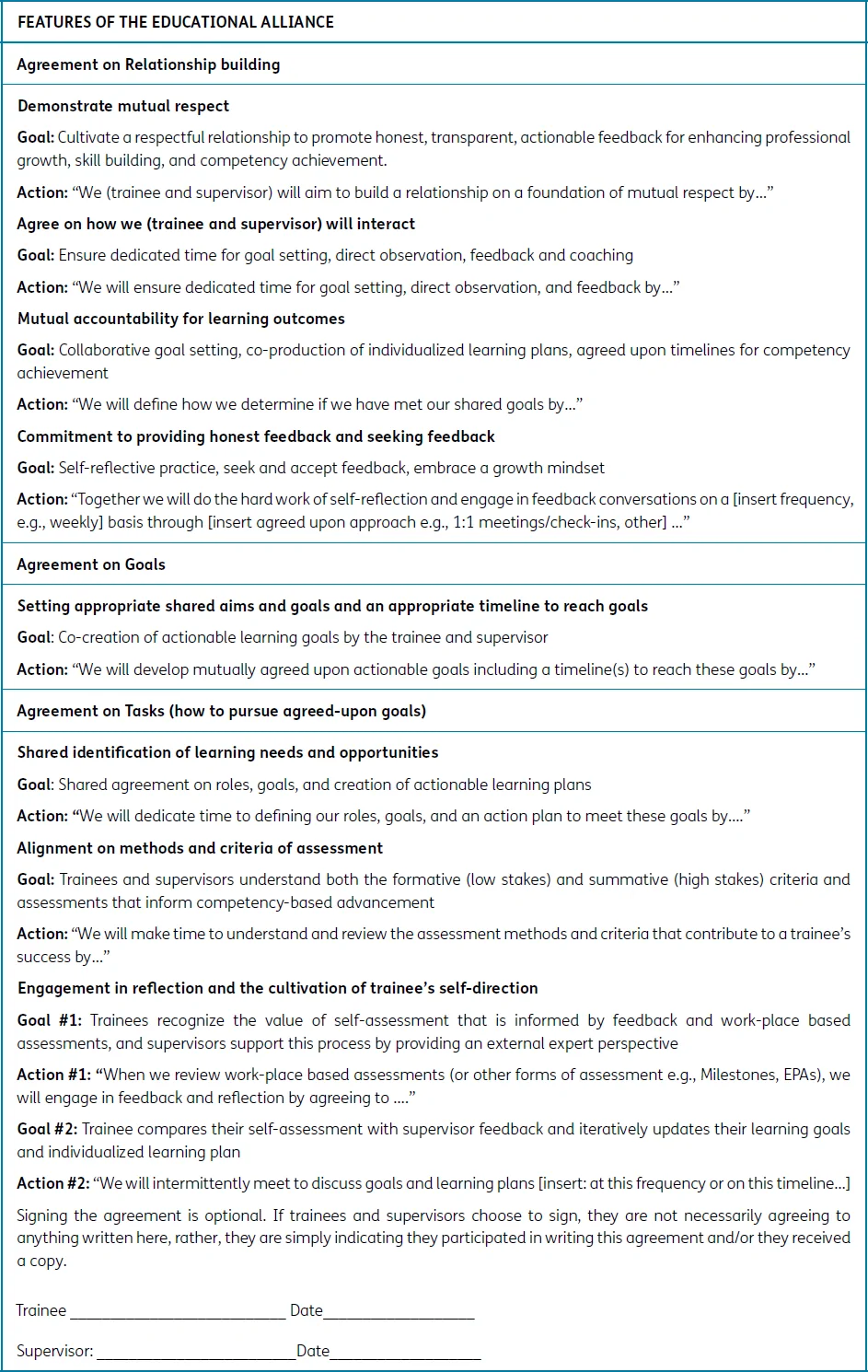
**An Educational Agreement to foster the educational alliance between trainee and supervisor.** This shared Agreement is centered on the three key features of the educational alliance: interpersonal relationship building, goals of engagement, and task orientation to achieve goals. Suggested goals are coupled with actionable “sentence starters” to facilitate trainee and supervisor dyads as they develop and strengthen their relationship through collaborative goal setting. This Agreement is a shared responsibility of both the educator and the learner. It is intended to be adapted to meet local needs and contexts. Trainees are defined as students, residents, fellows, or other learner(s) and supervisors are teachers, clinical teachers, faculty, or equivalent roles.

The Educational Agreement may be introduced during trainee orientation, supervisor retreats or regularly scheduled meetings. Program leaders should set expectations for use within local contexts, including suggestions for implementation (e.g., beginning, mid-rotation and end of rotation check-ins, coaching meetings). Local leaders may consider how to best implement the Agreement across contexts, roles, and settings, emphasizing voluntary participation and the overarching goal of promoting psychological safety within the educational alliance (Supplemental File 1: Appendix).

### Boundaries and Limitations of the Educational Alliance in Health Professions Education

Defining and respecting the boundaries of the educational alliance ensures that trainees and supervisors remain focused on advancing the trainee’s competence and professional identity formation. The educational alliance is a professional, goal-directed relationship grounded in trust, respect, and collaboration. Distinct from friendship, the educational alliance does not extend into trainees’ personal or social spheres. Unlike the therapeutic alliance from which it is conceptually derived, the educational alliance is not a clinical care relationship; the educational supervisor is not the trainee’s physician, health care provider, or therapist. The relationship is also time-limited and context-bound, focused specifically on advancing competence and professional identity formation rather than meeting broader personal needs. Confidentiality is expected, but with explicit limits where patient safety, institutional responsibilities, or policies require disclosure. Thus, the educational alliance, like the therapeutic alliance, preserves professional distance while fostering the conditions necessary for trust, openness, and meaningful competency-based learning.

The educational alliance may fail to form or be disrupted by structural tensions within clinical education. Supervisors often simultaneously occupy roles as coach and judge, and this dual responsibility can undermine trust if feedback is perceived as high-stakes or evaluative rather than developmental, complicating learners’ willingness to be vulnerable and engage authentically [42]. Alliances may also form unevenly, privileging shared identity, communication styles, or cultural alignment, with potential downstream inequities for minoritized trainees [41]. Conversely, a strong alliance may introduce risks of bias or leniency in assessment, underscoring the need for programmatic safeguards, multiple assessors, and transparent criteria to ensure that relational trust does not distort judgment and decision-making [43]. Further, alliances may occur in the context of more complex learning environments, particularly in educational settings where trainees are unionized and have dual roles as learners and employees [44].

A longitudinal relationship between a trainee and supervisor is the prototypical educational alliance and the focus of this manuscript. However, distinct types of educational alliances exist, including institutional relations between cohorts of trainees and whole faculties (Supplemental File 1: Appendix). While all comprise the elements of shared goals, agreement on how to achieve those goals, and a relational bond, these elements manifest differently based on relationship type. In addition, the distinction between any teaching interaction and an educational alliance relies on the trainee’s perception of the supervisor’s authenticity and commitment to the learner [24,45].

### Recommendations to Enhance the Educational Alliance within CBHPE Programs

Given the theory-supported importance of the educational alliance in supporting CBHPE, this manuscript proposes strategies for educators, program leaders, and trainees to cultivate and sustain effective alliances. These recommendations focus on fostering shared understanding, enhancing relationships, and supporting collaboration across all stakeholders. Recommendations span foundational concepts that can be incorporated into existing faculty development sessions and more aspirational directions.

#### Shared mental model

Building a shared mental model of the educational alliance and its core elements (relational bond, shared goals, and agreement on tasks to achieve goals) is foundational. Supervisor and trainee professional development programs should establish a common understanding of the educational alliance, emphasizing its central role in facilitating CBHPE. Training sessions should define the roles and goals of supervisors and trainees while exploring relevant learning theories [46], drivers of performance [47], and potential tensions in self-assessment and feedback-seeking behaviors [48]. Incorporating mindset theory [18] into these discussions provides further insight into the dynamics of growth and fixed mindsets, and the potential pitfalls of the “false growth mindset” in which trainees aim to appear to have a growth mindset [49]. Introducing these concepts helps normalize challenges within the trainee-supervisor relationship and learning environment, fostering honest communication and mutual understanding about what is working and what is not within the educational relationship [50].

#### Relationship-building

Supervisor development programs should focus on techniques for building trusting relationships, coaching methodologies, and evidence-based feedback practices. Supervisors play a crucial role in the educational alliance, and their training should include practical tools such as the R2C2 model for interactive feedback conversations that engage learners in reflection and improvement planning (Relationship building, Reaction exploration, Content exploration, Coaching for change) [48,51], along with guides and conversation starters to facilitate effective dialogue [52]. Supervisors should also be equipped to position themselves as credible sources of feedback, central to the alliance [2,26,48]. Training opportunities can be embedded within existing venues such as grand rounds, workshops, or microteaching sessions during faculty meetings, ensuring accessibility and integration into routine activities.

As the relational precondition for effective feedback, the educational alliance has similarities and distinctions from other important interactions in HPE. All supervisors work alongside learners and have a greater impact when they discuss feedback, instill trust, and cultivate psychological safety [53]. However, the educational alliance deepens focus through shared goals and a plan for working toward them inside a trusting relationship. By contrast, a mentor contributes advice-giving and role-modeling [54]. Coaching is the method of supporting personal development within a longitudinal, growth-oriented relationship [42,55].

#### Trainee agency

Trainee development should focus on cultivating agency through self-regulated learning, enabling co-production of learning, and encouraging effective feedback-seeking behaviors. Introducing the educational alliance concept during key transition points such as orientations to clerkships and core rotations can help trainees understand their role in building collaborative learning relationships. These sessions should emphasize the importance of taking the lead in goal setting, feedback conversations, and self-reflection to support their development in a competency-based environment.

#### Team training

Educational alliance training should also extend to interdisciplinary teams to strengthen team-based supervisory and trainee dynamics, recognizing that team training is still aspirational in some contexts. Incorporating educational alliance principles into team development initiatives can enhance relationships across disciplines and support junior supervisors as educators. This approach also fosters collaboration, encourages a growth mindset through continuous team improvement, enhances the clinical learning environment [56], and promotes involving trainees as teachers, further advancing CBHPE principles.

Flexibility in development program format is essential to meet the diverse needs of stakeholders. Joint training sessions with supervisors, trainees, and interdisciplinary team members can facilitate relationship-building across groups, while separate sessions by role or profession can create psychological safety and allow for discussions of group-specific concerns. Pairing teacher-trainee dyads in scenario-based exercises offers hands-on practice in applying educational alliance principles to real-world situations, preparing participants to navigate challenges effectively. Faculty development that focuses on developing the educator skills in alignment with CBHPE principles will complement these efforts [57].

#### Structured interactions

Intentional design of structured trainee-supervisor interactions is critical to fostering effective educational alliances in CBHPE programs. Early and ongoing clarification of expectations and roles helps build trust and align goals. Structured conversations at the relationship onset define objectives, establish mutual responsibilities, and create a shared understanding of what success looks like. Collaborative goal-setting further enacts the educational alliance, as trainees and supervisors co-create personalized learning goals that are specific, measurable, and aligned with both competency milestones and trainee interests. These goals should be revisited regularly to ensure alignment with the trainee’s developmental stage and emerging needs.

Regular, scheduled meetings embedded into the curriculum or program provide dedicated time for trainee-supervisor check-ins. While time for these meetings may seem aspirational, author experience suggests that the value added for both trainees and supervisors makes them feasible and desirable for participants. These meetings entail reviewing progress toward goals, addressing emerging learning needs, and adjusting strategies collaboratively. Documenting these discussions, goals, and action plans supports transparency, reinforces co-construction of the alliance, and helps track progress. Encouraging both trainees and supervisors to reflect on their interactions and the overall effectiveness of the alliance further strengthens the relationship and supports ongoing development.

#### Programmatic alignment

Programmatic structures aligned with educational philosophies and institutional priorities play a key role in fostering educational alliances within CBHPE programs. Clearly defined faculty roles (e.g., coach, advisor, or supervisor) that explicitly support the development and maintenance of educational alliances are essential. Programs can consider allocating dedicated educational time for faculty to foster trainee competency over time. These roles should align with program needs and CBHPE principles. Incorporating educational alliance responsibilities into faculty performance expectations for advancement and promotion recognizes and rewards efforts to establish strong relationships, engage in ongoing relationship-building, and collaboratively pursue mutual learning goals with trainees. Balanced with this recommendation is the need to avoid incentivizing faculty to ‘perform’ or appear to build an alliance rather than authentically engage in this relationship-building. Evidence from student evaluations of teaching demonstrates susceptibility to bias, power dynamics, and performative responding [58]. Avoiding individual-level data about the nuances of educational alliance interactions that could undermine trust and authenticity in the alliance is one solution to this challenge [59].

#### Conflict of interest

To ensure the integrity of the educational alliance, it is important to identify and mitigate conflicts of interest that may undermine trust. For example, supervisors who also hold roles as program directors or have influence over decisions related to trainee selection, grades, or awards should not concurrently exercise those evaluative functions within the same supervisory relationship as the educational alliance.

#### Transparent, ethical data use

Transparent and ethical data use is critical to maintaining trust within the educational alliance. Programs should establish policies that clearly define supervisor access to trainee data and specify how data may be used in decisions about trainee progression. Regularly collecting feedback from both trainees and supervisors about the learning environment can help programs assess whether institutional structures foster or hinder educational alliances and CBHPE principles.

These recommendations aim to strengthen educational alliances through supervisor, trainee, and team development strategies. By aligning these efforts with CBHPE principles, programs can cultivate mutual understanding, shared goals, and consistent engagement, ultimately advancing CBHPE.

### Areas for Future Research on the Educational Alliance in CBHPE

While the concept of the educational alliance is theoretically rigorous and holds promise for enhancing CBHPE, more empirical studies are needed to validate its efficacy and explore its practical implications in training environments. First, foundational research establishing conceptual clarity would distinguish between the educational alliance and teaching alliance, similar to Watling’s work differentiating feedback and assessment [42], to provide definitional precision and identify how alliance components drive successful educational outcomes. Developing a psychometrically sound instrument that captures educational alliance quality and its contribution to trainee competency development and wellbeing would elaborate on how the alliance quality influences educational outcomes [60]. Exploration of educational agreements, including how learning goals are negotiated and constructed between trainees and supervisors, would illuminate how dyads can best co-construct an enduring, effective alliance. Research qualitatively exploring trainee perceptions of feedback exchanges with supervisors and their goal-setting practices, viewed through the lens of growth mindset theory, could reveal how alliance-building behaviors shape learning. Leveraging existing large-scale datasets, such as from national organizations that collect data from multiple organizations, studies could examine associations between educational alliance presence and quality, trainee and patient outcomes, and broader institutional factors. Mixed methods studies that use such datasets and also incorporate qualitative approaches could elaborate why and how the educational alliance shapes experiences, perceptions, and outcomes. Longitudinal studies tracking how the alliance evolves across training stages and investigating its impact on both trainee and supervisor wellbeing would provide crucial insights into the relational dynamics that underpin effective, satisfying HPE. Finally, critical realism would serve as a literature-based frame for understanding the empirical (observed) data and actual experiences with the educational alliance, with the aim of uncovering underlying causal mechanisms (e.g., relational trust, credibility, psychological safety, identity affirmation) that produce effective feedback and learning under specific contextual conditions [61].

## Conclusion

The educational alliance, a trusting partnership between trainee and supervisor, facilitates the successful implementation of CBHPE through a mutual focus on feedback for growth and the promotion of self-regulated learning skills, with a steady focus on the defined outcomes of training. Programs can promote an educational alliance by establishing a shared mental model and providing training for trainees, supervisors, and teams on this important construct. Dedicated time for trainee-supervisor engagement, structured guidance for these interactions, and transparent procedures for using trainee performance information strengthen the effectiveness of the educational alliance for learning. Continued monitoring of the nature, strength and impact of educational alliances as part of program continuous quality improvement and research will contribute to successful competency-based education implementation.

**Supplemental File 1: Appendix:** Types of Educational Alliances within a Competency-Based Health Professions Education Program across relevant contexts, roles, and settings.


TRAINEE – SUPERVISOR RELATIONSHIP	CONTEXT	PURPOSE OF INTERACTIONS

Trainee and direct clinical supervisor	Regular (i.e., daily, weekly monthly) contact for finite period of clinical training assignment	Clinical supervision Feedback based on direct observation Focus on patient care tasks

Trainee and longitudinal coach, mentor, advisor	Often an assigned relationship that may last throughout the entire program	Longitudinal, developmental support Review of competency-based assessments and aggregate data Assessment of progress within the program Goal setting to achieve program outcome goals

Trainee and program director	Oversight over trainees within the training program	Focus on the integrity of the program (preventing malpractice, fostering inclusion)

Trainee and clinical competency committee	Periodic, scheduled review of progress toward program competencies and milestones	Making and justifying high stakes entrustment decisions

